# Carbon and energy balance of biotechnological glycolate production from microalgae in a pre-industrial scale flat panel photobioreactor

**DOI:** 10.1186/s13068-024-02479-4

**Published:** 2024-03-15

**Authors:** Heiko Wagner, Antonia Schad, Sonja Höhmann, Tim Arik Briol, Christian Wilhelm

**Affiliations:** 1 Department of Algal Biotechnology, Institute for Biology, University of Leipzig, Permoserstrasse 15, 04318 Leipzig, Germany; 2 Department of Solar Materials, Helmholtz Center for Environmental Research—UFZ, Permoserstrasse 15, 04318 Leipzig, Germany

**Keywords:** *Chlamydomonas*, Glycolate, Algae biotechnology, Volumetric productivity, Flat panel, Photobioreactor, Carbon sequestration

## Abstract

Glycolate is produced by microalgae under photorespiratory conditions and has the potential for sustainable organic carbon production in biotechnology. This study explores the glycolate production balance in *Chlamydomonas reinhardtii*, using a custom-built 10-L flat panel bioreactor with sophisticated measurements of process factors such as nutrient supply, gassing, light absorption and mass balances. As a result, detailed information regarding carbon and energy balance is obtained to support techno-economic analyses. It is shown how nitrogen is a crucial element in the biotechnological process and monitoring nitrogen content is vital for optimum performance. Moreover, the suitable reactor design is advantageous to efficiently adjust the gas composition. The oxygen content has to be slightly above 30% to induce photorespiration while maintaining photosynthetic efficiency. The final volume productivity reached 27.7 mg of glycolate per litre per hour, thus, the total process capacity can be calculated to 13 tonnes of glycolate per hectare per annum. The exceptional volume productivity of both biomass and glycolate production is demonstrated, and consequently can achieve a yearly CO_2_ sequestration rate of 35 tonnes per hectare. Although the system shows such high productivity, there are still opportunities to enhance the achieved volume productivity and thus exploit the biotechnological potential of glycolate production from microalgae.

**Supplementary Information:**

The online version contains supplementary material available at 10.1186/s13068-024-02479-4.

## Background

The sustainable production of organic carbon from CO_2_ is a major challenge for a climate friendly future global economy. Microalgae offer a promising approach for sustainable carbon fixation through the biotechnological production of glycolate using photorespiration [1]. Glycolate, a small C2 α-hydroxy acid, has great potential as a building block for numerous industrial products, such as in cosmetics [2], medicine, the textile industry, and as a household chemical due to its versatile properties as an alcohol and carboxylic acid. Additionally, glycolate can serve as a base chemical for the production of biodegradable and biocompatible polymers such as polyglycolic acid (PGA) or poly(lactic-co-glycolic acid (PLGA)) and for the formation of ethylene glycol [3, 4]. Traditionally, glycolate synthesis, e.g., through the carboxylation of formaldehyde, is derived from fossil raw materials and requires toxic compounds, raising concerns over sustainability and environmental impact [5]. The increasing industrial demand for glycolate has prompted extensive research into sustainable production methods. Consequently, the development of biotechnological approaches for glycolate production has garnered considerable interest. Microbial cell factories have been developed, particularly in *Escherichia coli* [6–8] and *Gluconobacter oxydans* [9]. Nevertheless, these technologies rely on organic carbon obtained from plant biomass, which competes with food production. To address these concerns and encourage a circular economy, considerable attention has been given to the use of photosynthetic microalgae for biotechnological glycolate production [1, 10–13]. In this study, the unicellular green alga *Chlamydomonas reinhardtii* was used as a model organism due to its well-described genetic background. Furthermore, the feasibility of genetic engineering and the availability of large mutant collections can aid in the analysis and optimization of metabolic pathways in the future.

Glycolate synthesis in *Chlamydomonas reinhardtii* is closely related to the photorespiratory pathway, a metabolic route linked to photosynthesis. In this process, oxygen (O_2_) and carbon dioxide (CO_2_) compete for the binding site on the ribulose-1.5-bisphosphate carboxylase/oxygenase (Rubisco) enzyme. Here, the oxygenation reaction of ribulose-1.5-bisphosphate leads to the formation of one molecule of phosphoglycerate and one molecule of phosphoglycolate. Phosphoglycerate can be reduced in the Calvin cycle to support the regeneration of ribulose-1.5-bisphosphate, whereas phosphoglycolate is metabolized in the C2 cycle. Since phosphoglycolate has a toxic effect on cells, green algae have the ability to excrete the molecule as glycolate in its dephosphorylated form during periods of increased photorespiration. This natural excretion process can be exploited biotechnologically for glycolate production without the need to engineer the biosynthetic pathway [11, 12].

Glycolate production is usually carried out in a two-step system [11, 12]. In the initial biomass production phase high CO_2_ levels are employed to promote rapid culture growth. Subsequently, in the glycolate production phase, the aeration conditions are adjusted to induce photorespiration, resulting in glycolate formation. During this second phase, the balance between carboxylation and oxygenation at Rubisco must be adjusted to a ratio of 2 to ensure adequate photosynthetic carbon fixation for glycolate production. For this purpose, a precise adjustment of the CO_2_:O_2_ ratio in the aeration flow of the bioreactor is crucial. Furthermore, when using *Chlamydomonas* wild-type strains, CO_2_ accumulation via carbon-concentrating mechanisms (CCM) [14] needs to be disrupted by adding inhibitors such as ethoxyzolamide (EZA). Alternatively, mutants with deficiencies in the CCMs or glycolate metabolism may be used for glycolate production [10, 13, 15].

Due to its direct photosynthetic origin, the efficiency of glycolate production is highly dependent on light conditions. It has been shown that adjusting factors such as light intensity and duration of irradiation can significantly enhance glycolate production [11]. A flat panel reactor equipped with artificial illumination is best suited for such a sophisticated setting. In contrast to larger tubular reactors and open ponds, the distinctive geometric structure of flat panel reactors, with its large surface-to-volume ratio and even dispersal of light throughout the culture medium, allows a high light use efficiency. In contrast, under natural lighting conditions, photoinhibition is a common challenge, especially during midday when irradiation levels are high [16].

In this context, the objective of the present study is to balance biotechnological glycolate production using *Chlamydomonas* in a modern flat panel photobioreactor at a demonstration scale. Optimal glycolate production rates are achieved by simultaneously monitoring parameters, including pH, dissolved O_2_ levels and supply of nutrients such as nitrogen (N). This enables the evaluation of the carbon and energy balance and provides crucial data for techno-economic analyses. The feasibility of a sustainable approach to producing renewable carbon backbones is highlighted.

## Methods

### Culture conditions and strain

Wild-type *Chlamydomonas reinhardtii* strain SAG 11-32b (SAG, Culture Collection of Algae, University of Göttingen) was grown on minimal medium based on a recipe for TP medium by [17]. The medium was modified by replacing the TRIS buffer with 20 mM MES (2-(N-morpholino)ethanesulfonic acid) and adding 25 µM Fe-EDTA along with trace elements from Bold’s Basal Medium (BBM) [18]. The pH was adjusted to 6.8 by addition of KOH. Batch cultivation at 100 µmol photons m^−2^ s^−1^ with a 14:10 h light:dark cycle at 25 °C was used for pre-cultures.

All culture conditions, ranging from pH to aeration, for both inoculation and PBR (photobioreactor) cultures, are based on previous evaluation of optimal conditions in small laboratory cultures [11]. Inoculation of the reactor chambers with pre-cultures resulted in final Chl *a* contents between 1 and 4 mg Chl *a* l^−1^. The two-phase production run started with a biomass growth phase followed by the actual glycolate production phase. During the initial biomass growth phase, the reactors were aerated with high CO_2_ levels (2%) and continuously exposed (continuous light) to 250 µmol photons m^−2^ s^−1^. After two days, the cultures were supplied with an additional 7 mM NH_4_^+^ to counteract nitrogen deficiency conditions, and grew until the Chl *a* content reached ~ 37 mg Chl *a* l^−1^. At this point, the glycolate production phase was initiated by adding 50 µM of the inhibitor 6-ethoxy-2-benzothiazolesulfonamide (EZA) dissolved in 50% ethanol (v/v). After an acclimation period of 1 h, the aeration of the cultures was switched to low CO_2_ (0.2%) and high O_2_ (35%) to induce photorespiration conditions.

### Chl content, cell number and dry weight determination

For chlorophyll determination, between 2 and 6 ml of culture were harvested onto a cellulose filter (MN 85/70, 25 mm, Macherey–Nagel, Düren, Germany). A homogenizer (Precellys Evolution, Bertin Technologies, France) was used for pigment extraction. Cells were digested in 2 ml of 80% acetone (v/v), using a 3:1 mixture of glass beads with a diameter of 0.25–0.5 mm and 0.75–1 mm. After centrifugation (2 min, 14,000*g*), absorbance was determined at wavelengths of 664 and 647 nm. Chl a concentrations were calculated according to the method of [19].

To determine cell numbers, between 500 μl and 2 ml were diluted with 10 ml isotone. Cells were counted using an automatic cell counter (Z2 Coulter Particle Count and Size Analyzer, Coulter Electronics Inc. Miami, USA).

To determine the dry mass, 20 ml of algal culture was removed from the reactor and centrifuged at 2000 ×*g* for 5 min. The pellet was washed with distilled water, centrifuged again, resuspended in 2 ml distilled water and transferred to a weighed 2 ml reaction vessel. The cells were pelleted at 1000 ×*g* for 3 min and freeze-dried. The dry weight was then balanced to determine the weight per cell. The biomass concentration of the culture was subsequently calculated as biomass [mg l^−1^] via the cell count.

### Photobioreactor design

The scalable photobioreactor design was planned and implemented in collaboration with Prof. C. Posten from Karlsruhe Institute of Technology (KIT). A previously described custom-built 10 l flat panel bioreactor served as a model [20]. The final flat panel reactor comprised three identical compartments (Additional file 1: Fig. S1; Table S1 for detailed description). The culture chamber of each compartment was constructed using two borosilicate glass plates with dimensions of 55.6 cm × 66.7 cm and a thickness of 6.6 mm. This specific size was selected to minimize pressure-induced glass deformation while achieving a 1 m^2^ exposed reactor area across the three compartments. The glass panels were designed to be fully disassembled, allowing to adjust the layer thickness of the resulting culture chambers from 2 up to 20 mm using different spacers. For the experiments presented here, the layer thickness was set to 10 mm, thus, when fully filled, the total volume of one reactor compartment corresponded to 3.3 l. Illumination was provided by a LED light panel placed at a distance of 50 cm on one side of the photobioreactor (Additional file 1: Fig. S1A), equipped with 143 single LEDs with a broad white emission spectrum (Additional file 1: Fig. S2).

**Fig. 1:**
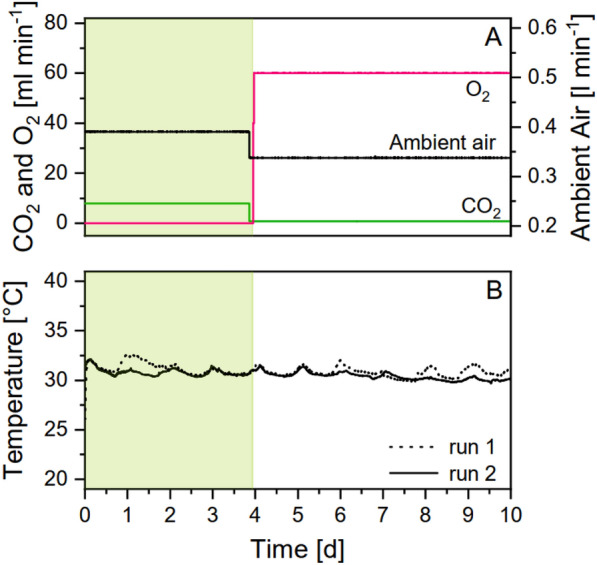
Measurement parameters recorded by the reactor. Parameters are shown for two runs (run 1: dotted line; run 2: solid line). Gas composition could be precisely adjusted and controlled for CO_2_, O_2_ and ambient air (**A**). Temperature (**B**)

Mixing and carbon supply were achieved by bubbling the chamber with gas of an adjustable composition. The gas flow was controlled separately for CO_2_, O_2_ and air, by a corresponding mass flow controller (MKS, Munich, Germany). The gas mixture was fed into the medium through 6 syringe needles (0.3 mm diameter, Sterican, B. Braun, Germany) at the bottom of the reactor chamber, directing the airflow through the complete reactor. The composition of the gas inflow was monitored during each run (Fig. 1A), with the total flow rate remained constant at 400 ml min^−1^. During the biomass phase, ambient air was mixed with CO_2_, at flow rates of 392 ml min^−1^ and 8 ml min^−1^ respectively, to achieve CO_2_ concentrations of 2%. For the glycolate phase, the flow rate of CO_2_ was reduced to 0.8 ml min^−1^, while O_2_ was added at a flow rate of 60 ml min^−1^. This corresponded to 0.2% CO_2_ and 35% O_2_ in the medium.

Thermoregulation was provided by a second, adjacent chamber of equal dimensions through which tempered water was circulated. The temperature was measured in the pump water system and inside the reactor chamber, respectively. The pump control system was connected to a thermostatic bath (Julabo F-12; Julabo, Germany). Water temperature was manually adjusted, depending on the measured temperature inside the reactor. Thus, the temperature inside the reactor was largely controlled around 30 °C (Fig. 1B). Although the reactor was placed indoors in an air-conditioned room, temperature readings showed a slight day–night influence. Maximum temperatures during the day reached 33 °C, while during the nights a minimum of 30 °C was measured.

Two sensors were placed through specially designed ports in the glass to measure pH and dissolved O_2_ inside the reactor. pH was adjusted by a computer controlled pump that feeds KOH into the reactor chamber.

The control and data acquisition of the individual reactor parts (gassing, LED lighting, temperature, pH, etc.) was carried out via a BioProCon process control system based on LabView (National Instruments), which was developed and implemented previously [20]. It was specially adapted to the reactor system and allowed simultaneous, independent control of the three separate reactor chambers.

At the beginning of each run, the reactor system was chemically sterilized by flooding the chamber and attached tubing with 0.05% H_2_O_2_ for 24 h. The system was then rinsed twice with sterile distilled water before the chamber was filled with the pre-culture as described above.

### Glycolate determination

To determine the glycolate concentration in the reactor medium, 1 ml of the culture was centrifuged (2 min, 12.100 ×*g*), the supernatant was filtered (PES syringe filter, 0.2 µm pore size) and stored at – 20 °C. HPLC analysis was performed using the UltiMate 3000 HPLC system (ThermoFisher Scientific, USA) equipped with a Hi-Plex H ion exchange column (300 mm × 7.7 mm, Agilent Technologies, USA). For separation, 10 µl of the samples was applied to the column. The mobile phase was 5 mM H_2_SO_4_ in milliQ H_2_O. Isocratic elution was performed at 15 °C, at a flow rate of 0.75 ml min^−1^ for 20 min. For calibration, standard solutions of known glycolate concentrations in minimal medium were measured. Glycolate was detected by UV–Vis spectroscopy at 210 nm (VWD-3100 detector). The concentration in the sample was calculated from the integrated peak area using Chromeleon software (ThermoFisher Scientific, USA).

### NH_4_^+^ determination

Anion concentrations inside the medium were measured by ion chromatography using a Dionex Integrion HPIC System (ThermoFisher Scientific, USA) equipped with a CS19 column. Culture samples were prepared as described above for chromatographic glycolate determination. After filtration, samples were diluted 1:200 with milliQ H_2_O. The eluent produced by the EGC MSA (methanesulfonic acid) cartridge was applied in a gradient with the following steps: 0–15 min at 3 mM, 15–20 min: increase to 15 mM, 22–23 min: decrease to 3 mM, 23–27 min: 3 mM. The flow rate was 0.25 ml min^−1^ at 30 °C. For calibration, Dionex Six Cation-II standard solution was used to prepare samples with known concentrations.

## Results

### Controlling chemical and physical parameters in the flat panel reactor

**Fig. 2:**
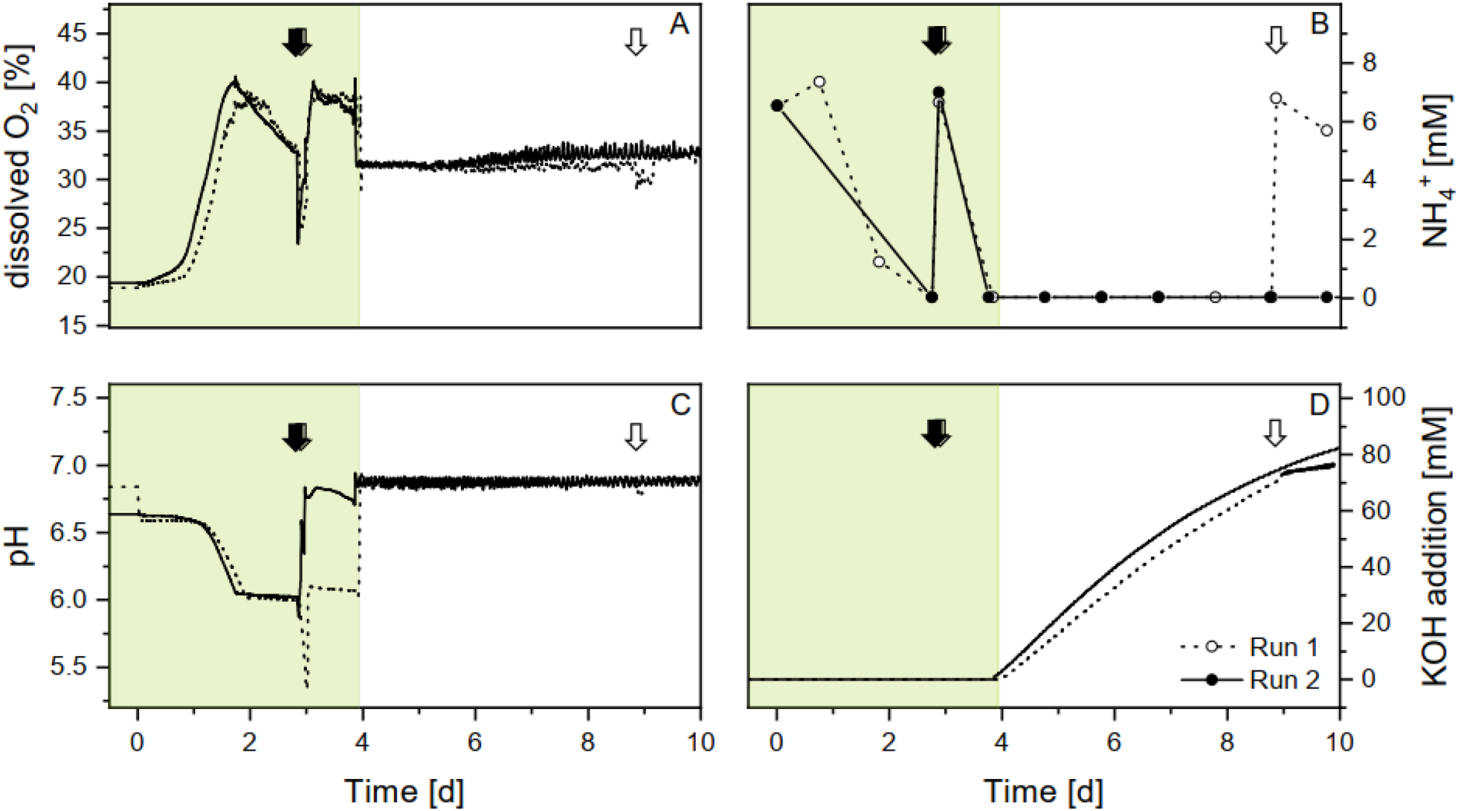
Measurement parameters recorded by the reactor. Parameters are shown for two runs. During the biomass phase (green shading), cultures were grown with 2% CO_2_ in ambient air. Glycolate production was induced by switching aeration to 0.2% CO_2_, 35% O_2_ in ambient air. Arrows mark the time point of additional 7 mM NH_4_^+^ supply. **A** Internal O_2_ concentration. **B** NH_4_^+^ concentration in the reactor medium measured by ion chromatography. **C** The pH of the culture controlled by **D** the activity of an automatic pump that added KOH when the medium acidified

**Fig. 3:**
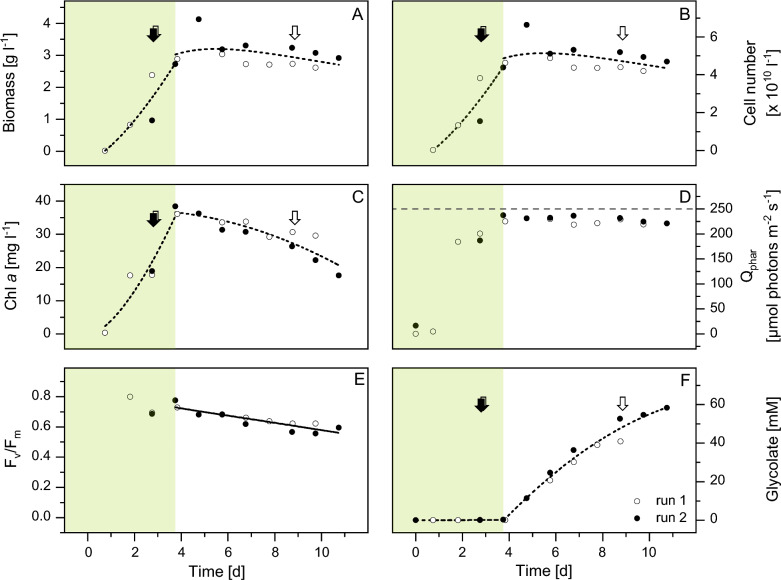
Growth and glycolate production of *Chlamydomonas reinhardtii* in a flat panel reactor. Biomass (**A**), cell numbers (**B**), Chl *a* concentration (**C**), Q_Phar_ (**D**), F_v_/F_m_ (**E**), and glycolate accumulation (**F**) of two independent runs (run 1: open symbols; run 2: closed symbols). During the biomass phase (green shading), cultures were grown with 2% CO_2_ in ambient air. Glycolate production was induced by switching aeration to 0.2% CO_2_, 35% O_2_ in ambient air. Arrows mark the time of additional 7 mM NH_4_^+^ supply. The horizontal dashed line in (**D**) indicates the irradiated photosynthetically available radiation (PAR) of 250 µ mol photons m^−2^ s^−1^

From the beginning of each run, K^+^ and NH_4_^+^ ion concentrations were monitored externally, whereas parameters such as dissolved oxygen concentration and pH were recorded within the reactor by several sensors and the corresponding software. As no additional O_2_ was supplied to the reactor during the biomass phase, the dissolved oxygen concentration was initially at around 19%, as found in ambient air (Fig. 2A). With advanced biomass growth (Fig. 3A) and increasing photosynthetic activity within the first two days, the dissolved oxygen concentration started to rise towards a maximum of 40%. Subsequently, the dissolved oxygen concentration decreased steadily to a value of about 35% on the third day, indicating a slightly reduced photosynthetic activity compared to the optimum (Fig. 2A). The measured NH_4_^+^ concentration initially corresponded to the 7 mM added to the medium, but was completely depleted within the first 3 days. In response to the decreasing photosynthetic activity, an additional 7 mM NH_4_^+^ was added to the medium (Fig. 2B) to ensure sufficient nutrient supply during biomass formation phase. This addition of NH_4_^+^ resulted in a rapid drop in dissolved O_2_ to 25% in both runs, and a concurrent decrease in pH (Fig. 2C).

However, for the stability of the culture, the pH should be kept at an optimum of around 6.9 and large fluctuations should be avoided. Initially, the pH was adjusted between 6.6 and 6.9. It gradually dropped to pH 6 in both runs during the first 3 days. As the addition of 7 mM NH_4_^+^ caused a further drop in pH, it was manually raised by the addition of KOH. In run 1, the pH was adjusted to pH 6 for the remaining biomass phase and to pH 6.9 with the beginning of the glycolate production phase. However, in run 2, the pH was adjusted to pH 6.9 immediately after NH_4_^+^ addition. Subsequently, the oxygen concentration increased again to the maximum of 40%, where it remained constant until the end of the growth phase. This can be explained as a result of increased photosynthetic activity due to the improved nitrogen supply. In contrast, the different pH values during the remaining biomass phase had no effect on oxygen production, cell growth or Chl *a* content (Fig. 3B, C). Due to the increased biomass growth, the newly added nitrogen during the biomass phase was assimilated by the biomass within a very short time. Even after this addition, no NH_4_^+^ could be detected after a subsequent 24 h of biomass growth. During the glycolate phase, the reactors continued to operate under these nitrogen-depleted conditions.

After the change in gas composition at the beginning of the glycolate phase, the dissolved oxygen in the medium immediately dropped to around 30%, indicating a decrease in photosynthetic O_2_ production. It appears that the dissolved oxygen concentration was mainly controlled by the externally applied gas flow adjusted to 35% O_2_, as its value remained fairly constant at 31% and increased to 34% at the end of the glycolate production phase. An additional application of NH_4_^+^ in run 1 on day 9 had no significant effect on oxygen levels.

The pH was manually adjusted to about 6.9 at the beginning of the glycolate phase and maintained at 6.9 by a KOH pump during glycolate production (Fig. 2D). As a result, the KOH concentration increased more rapidly and then levelled off slightly over time. The final calculated concentration of KOH added during the entire glycolate phase was between 76 mM (run 1) and 82 mM (run 2).

### Culture growth and subsequent glycolate production in the flat panel reactor

During the initial four-day biomass phase, the cultures were aerated with CO_2_ concentrations of 2%. As a result, both cultures showed rapid growth at the beginning of the run. Total biomass reached its maximum value of 3.57 g dry weight l^−1^ around day 4–5 (Fig. 3A). Cell numbers, which were used together with the dry weight per cell for biomass calculation, steadily increased in the same pattern as biomass, up to 4.5 × 10^10^ cells l^−1^ in both runs (Fig. 3B). Chl *a* initially increased to about 18 mg l^−1^ (Fig. 3C) at the end of the second day, but after the addition of 7 mM NH_4_Cl, this amount doubled to a maximum of about 37 mg l^−1^ by the end of the third day. Thus, nitrogen fertilization had a lesser impact on the increase in cell number than on the rise of Chl* a* content in the cultures.

Shortly after switching to glycolate-producing conditions, the total biomass decreased slowly, reaching a final value of 2.76 g dry weight l^−1^ towards the end of the run. At the same time, a decrease in Chl *a* concentration was observed. At the end of the run on day 10, Chl *a* concentrations dropped below 20 mg l^−1^. However, the cell concentrations remained in a similar range as before. Therefore, the chlorophyll content per cell decreased continuously during the glycolate production phase. As the cells did not bleach out completely, they still retained their photosynthetic and physiological activity.

Photosynthetically absorbed radiation (Q_Phar_) was calculated by recording daily Chl *a* content and the absorption spectrum of the culture (Fig. 3D). Initially, when the Chl *a* content of the culture was low, Q_Phar_ also showed low values between 0 and 25 µmol photons m^−2^ s^−1^. With the rapid increase in Chl *a* concentration, Q_Phar_ also increased from the second day, reaching a maximum approaching the fourth day in both runs, with 225 µmol photons m^−2^ s^−1^ (run 1) and 237 µmol photons m^−2^ s^−1^ (run 2). This corresponded to 90 or 95% of the irradiated light of 250 µmol photons m^−2^ s^−1^. After the reactor was transferred to the glycolate production phase, Q_Phar_ maintained these high values until the end of both runs, although the Chl *a* content decreased slightly during this period (Fig. 3A).

At the beginning of the biomass phase, F_v_/F_m_ was optimal, reaching values of 0.8 in run 1 (Fig. 3E). As the biomass phase progressed, the value continued to decrease, attaining a minimum of 0.7 in both runs at the end of day 2. After addition of 7 mM NH_4_^+^, F_v_/F_m_ briefly increased again in both runs to 0.78 (run 1) and 0.73 (run 2). However, following the induction of the glycolate phase, the value decreased continuously. Towards the end of the ten days, values of 0.59 (run 1) and 0.62 (run 2) were recorded. Clearly, in the state of continuous glycolate production, about 80% of the photosynthetic capacity is maintained.

From the time of glycolate induction, glycolate increased continuously in the medium (Fig. 3F). On day 10, 58 mM (run 1) and 54 mM glycolate (run 2) were measured. To further test the effects of additional nitrogen supply on glycolate excretion and growth parameters, we applied an additional nitrogen pulse during the glycolate phase (day 9 of the whole process) in run 1, while maintaining a constant nitrogen limitation in run 2 (Fig. 2B). However, no significant effect on glycolate production has been observed (Fig. 3F). Although the increase in glycolate flattened slightly towards the end of the observation period, this was associated with a bacterial contamination as the decrease occurred in both reactors and was not concurrent with NH_4_^+^ addition. This lower production of glycolic acid was also evident from the fact that the consumption of pH-balancing KOH decreased towards the end of the production period (Fig. 2D).

### Carbon and energy balance

The efficiency of carbon fixation can be expressed in terms of carbon balance, with the overall process proving to be an effective carbon sink. The maximum biomass production up to the beginning of the glycolate phase was 3.57 g dry weight l^−1^. However, during glycolate production, biomass decreased to a minimum of 2.76 g dry weight l^−1^. The biomass loss during the glycolate production phase, i.e., the difference between maximum and minimum biomass was, therefore, 0.81 g dry weight l^−1^ (Table 1). During the same glycolate production period, about 4.25 g l^−1^ of glycolate was produced. Additionally, all values were extrapolated to a theoretical industrial plant with 1 ha irradiated reactor surface. It was assumed that 10,000 reactor compartments of 1 m^2^ each are available for production over the course of 1 year. Based on conservative estimates and accounting for maintenance times, it is reasonable to anticipate 30 runs per year. Therefore, an annual production of approximately 13 t of glycolate is expected. At the same time, 8.4 t of residual biomass can be expected (Table 1), which can be used for further processing.

**Table 1:** Carbon balance on the basis of measured data in the 1 m^2^ reactor

	Measured values [l^−1^ reactor run (10 days)^−1^]	Upscaled values [ha^−1^ reactor surface y^−1^]
total amount of CO_2_ gassing (whole process)	15.95 ± 0.1 [l]	95.4 ± 0.6 [t]
biomass as dry weight (start of glycolate production)	3.57 ± 0.8 [g]	10.9 ± 2.3 [t]
biomass as dry weight (end of production)	2.76 ± 0.2 [g]	8.4 ± 0,6 [t]
biomass loss as dry weight through glycolate production	0.81 ± 0.6 [g]	2.5 ± 1.7 [t]
glycolate content (end of production)	4.25 ± 0.2 [g]	13 ± 0.6 [t]
total amount of CO_2_ gassing (whole process)	713 ± 4.6 [mmol]	2167.4 ± 14 [kmol]
gassing amount of CO_2_ (biomass phase)	596 ± 9.2 [mmol]	1813 ± 28 [kmol]
gassing amount of CO_2_ (glycolate production phase)	117 ± 13.8 [mmol]	354.4 ± 42 [kmol]
C stored in biomass (start of glycolate production)	148.6 ± 31.5 [mmol]	451.8 ± 96 [kmol]
C stored in biomass (end of glycolate production)	114.8 ± 8.5 [mmol]	349.2 ± 26 [kmol]
C loss from biomass through glycolate production	33.8 ± 23 [mmol]	102.7 ± 70 [kmol]
C content of glycolate (end of production)	112.6 ± 4.8 [mmol]	342.5 ± 15 [kmol]
C fixed in (biomass + glycolate)/CO_2_ injected (whole process)	31.9 ± 1.7%	
C fixed in biomass/CO_2_ injected (biomass phase)	25 ± 5.7%	
C fixed in biomass/CO_2_ injected (glycolate production phase)	− 5.7 ± 4%	
C fixed in glycolate/CO_2_ injected (glycolate production phase)	97.1 ± 7.4%	

To achieve these values, each litre of reactor volume had to be supplied with 15.95 l of CO_2_ (713 mM CO_2_) for an entire process cycle (biomass phase and glycolate production phase) (Table 1). This corresponded to 596 mmol C l^−1^ reactor volume during the biomass phase (2% CO_2_) and 117 mmol C l^−1^ for the glycolate production phase (0.2% CO_2_). Of the 596 mmol C l^−1^ supplied during the biomass phase, 25% or 148.6 mmol C l^−1^ was initially stored in the biomass, assuming that 50% of the biomass is carbon [21].

In contrast, during the glycolate production phase, 112.6 mmol l^−1^ of the supplied 117 mmol C l^−1^ was fixed in the form of glycolate, which accounted for 97% of the carbon. Biomass degradation during the glycolate production phase resulted in a C loss of 33.8 mmol C l^−1^, so that the C content of the remaining biomass at the end of the run was 114.8 mmol C l^−1^. Overall, the carbon balance in the two phases showed that 31% of the total supplied CO_2_ was bound in glycolate and biomass in one complete run. These data were then used to calculate the required C supply and its fixation rate of a hypothetical production plant with 1 ha illuminated reactor surface per year (Table 1). CO_2_ gassing of 95.4 t ha^−1^ y^−1^ of CO_2_ would be required, of which 29.6 t ha^−1^ y^−1^ of CO_2_ will be captured either in the glycolate product or in the remaining biomass. The system would, therefore, act as a CO_2_ sink.

The balance finally leads to a volumetric productivity for C fixation (*VP*_*C*_) of *VP*_*C*_ = 14.6 mg carbon l^−1^ h^−1^ in the biomass during the biomass phase (Table 2). During the following phase of glycolate production, *VP*_*C*_ in the glycolate was measured at 8.8 mg C l^−1^ h^−1^. Thus, the absolute C fixation in biomass and glycolate within one year is *VP*_*C*_ = 6.21 mg C l^−1^ h^−1^ and *VP*_*C*_ = 4.68 mg C l^−1^ h^−1^ respectively, assuming 30 cycles of 10 days each, consisting of ~ 4 days biomass phase and ~ 6 days glycolate phase in each cycle.

**Table 2:** Volume productivity for carbon fixation (*VP*_*C*_) and product formation (*VP*_*Glycolate*_). *VP*_*C*_ in biomass is calculated for ~ 4 days of the biomass phase and *VP*_*C*_ in glycolate for ~ 6 days of the glycolate phase. The volume activities are calculated for one run and for one year assuming 30 cycles of 10 days each, which consist of ~ 4 days biomass phase and ~ 6 days of glycolate phase in each cycle

	Volume productivity	
	1 run	1 year (30 runs)
*VP*_*C*_ in biomass	14.6 ± 5.02 [mg C l^−1^ h^−1^]	6.2 ± 1.35 [mg C l^−1^ h^−1^]
*VP*_*C*_ in glycolate	8.7 ± 0.67 [mg C l^−1^ h^−1^]	4.7 ± 0.2 [mg C l^−1^ h^−1^]
*VP*_*Glycolate*_	27.7 ± 2.14 [mg Glycolate l^−1^ h^−1^]	14.8 ± 0.61 [mg Glycolate l^−1^ h^−1^]

The volume productivity of product formation (*VP*_*Glycolate*_) was 27.7 mg glycolate l^−1^ h^−1^ during the glycolate production phase (Table 2). Assuming 30 production cycles per year, this means that glycolate can be produced at a rate of *VP*_*Glycolate*_ = 14.83 mg l^−1^ h^−1^ within one year.

Using flat panel reactors, not only mass balances can be estimated, but also energy consumption based on quantum efficiencies. During one run, the reactor was continuously irradiated with 250 µmol photons m^−2^ s^−1^. With a duration of 10 days per run, the available incident light (Q_PAR_) amounts to a total of 22.14 mol photons m^−2^ (Table 3). Using high-efficiency LEDs with 40% yield, the energy required to generate this amount of light is 3.37 kWh for one reactor run. The part of the photons actually absorbed for photosynthesis (Q_Phar_) can be calculated using the Chl *a* specific in-vivo absorption coefficient a^*^ [22]. Over the entire course of a run, Q_Phar_ reaches an average value of 17.06 mol photons m^−2^, which means that 77% of the incident light is absorbed by the culture. From these values and the amount of carbon bound (Table 1), the amount of photons required for each mol of C bound in biomass or glycolate can be determined. This quantum efficiency of carbon assimilation (φC) was 74.94 photons mol (mol C)^−1^ for the total process. For the up-scaled process for a reactor area of 1 ha under the same conditions and over a time period of one year, this means an energy demand of 10.3 GWh (Table 3).

**Table 3:** Light parameter, energy demand and photon use efficiency (φC) of the entire process. The energy demand for illumination is derived from Q_Par_, assuming an LED efficiency of 40% and a conversion factor of 4.56 J per photon. φC is calculated from the absorbed photons Q_Phar_ and the total C stored in biomass and glycolate at the end of production (Table 1)

	Measured values [l^−1^ reactor run (10 days)^−1^]	Upscaled values [ha^−1^ reactor surface y^−1^]
Incident light (Q_PAR_)	22.14 ± 1.5 mol photons	6.7*10^7^ ± 4.6*10^6^ mol photons
Absorbed photons (Q_Phar_)	17.06 ± 1.6 mol photons	5.2*10^7^ ± 4.9*10^6^ mol photons
Energy for incident light*	3.37 ± 0.2 KWh	10.3 ± 0.7 GWh
φC (Biomass phase)	29.84 ± 7.1 [mol photons mol C^−1^]	
φC (C fixed in glycolate)	112.91 ± 10.8 [mol photons mol C^−1^]	
φC (overall process)	74.94 ± 2.8 [mol photons mol C^−1^]	

## Discussion

### The role of nitrogen in the glycolate production system

Before the glycolate production was started, a high-density biomass culture was grown to a maximum biomass of 3.57 g dry weight l^−1^. The rapid reduction of nitrogen in the medium during this growth phase can be attributed to culture growth, since nitrogen is essential for the increase of cell numbers and Chl *a*. To prevent N-limitation, additional N was supplied, resulting in a total of 14 mM NH_4_^+^. Assuming that the carbon content of the biomass is 50% [21], this results in a maximum C:N ratio of 10.6, if the supplied nitrogen is completely taken up by the cells. Under adequate nitrogen supply, the C:N ratio in *Chlamydomonas* should be about 6, although it can increase to 14 under nitrogen deficiency [23]. It can, therefore, be assumed that the cells are already N-limited despite the addition of nitrogen. This conclusion is supported by the observed steady decrease in photosynthesis with increasing nitrogen deficiency, which has been reported previously [24, 25].

While sufficient nitrogen availability during biomass growth is essential for the formation of proteins, pigments and nucleic acids, a nitrogen deficiency in the glycolate phase may have certain advantages. Nitrogen deficiency should inhibit the C2 cycle, in which glyoxylate, the downstream product of glycolate, is further converted to the amino acid glycine. This reaction requires an NH_2_ group from alanine or glutamate as a donor [26, 27]. Nitrogen deficiency, therefore, limits the availability of donors and favours the accumulation of large amounts of glycolate in the cell. These elevated glycolate concentrations must be detoxified by excretion and consequently lead to accelerated glycolate accumulation in the medium. In this study, however, additional nitrogen supply during the glycolate phase in run 1 had no significant effect on glycolate production (Fig. 3F). In contrast to the biomass phase, where nitrogen was quickly depleted, the nitrogen added during the glycolate production phase declined more slowly and was still detectable after 24 h. Along with this, no changes in biomass growth were observed with the addition of N.

In this study, NH_4_^+^ was used as the preferred nitrogen source for *Chlamydomonas* due to its reduced state and, therefore, energetically favourable assimilation. Thus, the electrons obtained from the light reaction, which are normally required for the reduction of other nitrogen species (i.e., NO_3_^−^), are available for the photosynthetic reduction of CO_2_. This increased CO_2_ fixation usually leads to higher growth rates during the growth phase [28]. During the glycolate phase, however, only small amounts of nitrogen are required because biomass growth is strongly inhibited since the assimilated carbon is invested in glycolate excretion rather than cell proliferation. The amount of nitrogen, therefore, plays only a marginal role.

In *Chlamydomonas*, ammonium is the preferred N source for growth [29] and was, therefore, used for this study. However, it is well known that nitrate is also an efficient N donor in *Chlamydomonas reinhardtii,* with important impacts on physiological performance. First, NO_3_^−^ is an additional electron sink and can reduce carbon assimilation. Second, nitrate assimilation depends on a complex regulatory network of transporters and enzymes located in different cellular compartments [30]. Third, nitrate assimilation increases the pH during growth, in contrast to the decreasing pH during ammonium assimilation [31], so the pH control needs to be adjusted. Such an increase in pH may also be beneficial as it counteracts the acidification of the medium caused by glycolate production, thus reducing the amount of added KOH in the glycolate phase. As an alternative, the use of NH_4_NO_3_ as nitrogen source, which combines the different properties of NH_4_^+^ and NO_3_^−^, may be advantageous [32]. Further studies on the cellular degree of reduction and an optimal stoichiometry between the different nitrogen species could help to stabilize the system.

In conclusion, nitrogen is a critical component in biotechnological batch processes for glycolate production. In the future, pathway analysis can help to further elucidate the importance of nitrogen in glycolate metabolism, for example, using mutants of N-related pathways together with sophisticated metabolomics analysis. However, it is clear that monitoring nitrogen levels in batch processes is essential and that the amount of nitrogen should be carefully calculated to ensure optimum performance.

### Dissolved oxygen pattern during whole cultivation

The oxygen concentration in the reactor is influenced by various overlapping biological and physical processes. During the biomass phase, the concentration of externally added oxygen accounts for 19%, corresponding to the atmospheric oxygen content. However, the growing biomass with high photosynthetic activity causes the oxygen concentrations in the medium to increase up to 40%. The drop in oxygen that follows the addition of nitrogen is not pH dependent, as the O_2_ decrease was observed in both runs, but only in one run the pH dropped (in the other run it was counteracted with KOH) (Fig. 2). Thus, the conclusion is obvious that the oxygen drop is caused by the short-term high NH_4_^+^ concentrations. Indeed, studies on chlororespiration have shown that 5 mM NH_4_^+^ uncouples the proton gradient across the thylakoid membrane [33]. This uncoupling very quickly leads to an ATP bottleneck, consequently inhibiting the photosynthetic electron transport and O_2_ production. The large nitrogen deficit and the high biomass loading apparently result in a rapid removal of excess NH_4_^+^ within a short time (30 min), and the original oxygen rates of 40% can be restored (Fig. 2a).

It is assumed that oxygen inhibition starts to occur above 40%, which easily can be reached in dense and fast growing cultures, especially in closed PBRs [34]. Such high oxygen is not desirable, as they can accompanied by a decrease in biomass productivity and microalgal growth [35]. In this study, however, such high oxygen concentrations do not seem to influence growth and photosynthesis. After the initial rise to 40%, oxygen concentrations subsequently dropped rapidly to 33%. This decrease occurred at the same time as nitrogen in the medium was almost completely depleted. This suggests that the decrease in oxygen production is primarily due to reduced photosynthetic activity caused by nitrogen deficiency and probably not due to the harmful oxidative stress. Oxidative stress can lead to processes such as photoinhibition, since it is accompanied by the production of reactive oxygen species (ROS) such as singlet oxygen (^1^O_2_), superoxide radicals (O2^•–^) or hydrogen peroxide (H_2_O_2_). Due to their highly reactive nature, they can damage cellular components i.e., membrane lipids, DNA and photosystem II (PSII) [36]. ROS are formed when the excited PSII is no longer able to discharge electrons via the electron transport chain to a suitable acceptor, and instead the electrons are transferred to oxygen. To prevent this, *Chlamydomonas* has developed several mechanisms to distribute or dissipate the absorbed light energy. This can be achieved, for example, through state transitions, where a fraction of the PSII outer antenna is transferred to PSI, balancing the excitation energy in both photosystems [37]. Other mechanisms for the redistribution and dissipation of excessive energy within the PSII include zeaxanthin-dependent heat dissipation (NPQ) via the xanthophyll cycle, or the use of alternative electron transport pathways [38, 39].

However, the maximum efficiency of PSII is unchanged, as no significant decrease in F_v_/F_m_ levels is observed during this time. This indicates that the structure of the PSII is still largely preserved, which is consistent with the finding that short-term oxidative stress with low levels of ROS has no detectable effect on photosynthetic efficiency in *Chlamydomonas*. However, higher levels of ROS would decrease F_v_/F_m_ levels and inhibit photosynthetic efficiency [40]. *Chlamydomonas* possesses an efficient scavenging system for ROS and uses the increasing presence of ROS as a signal to adapt to high oxygen stress [41]. This means that *Chlamydomonas* can tolerate these short-term elevated oxygen levels caused by its own photosynthetic activity.

Despite the high concentration of dissolved O_2_ during the biomass phase, no glycolate excretion could be detected. There are two main reasons for this: First, the addition of CO_2_ increases the C:O ratio and thus counteracts photorespiration. Under such optimal CO_2_ supply the rate of photorespiration is drastically reduced. Secondly, any glycolate produced is quickly metabolized in the C2 cycle. The released carbon is recovered and fed to the carboxylation reaction at the Rubisco.

This scenario changed with the change in gassing and the addition of the inhibitor. The O_2_ partial pressure was kept high above 30%. However, the C:O ratio changes drastically, making photorespiration much more pronounced, and because CCMs are inhibited, an increase in glycolate is observed. However, F_v_/F_m_ decreases only slightly, indicating that there is no massive accumulation of ROS and that the effects of photoinhibition on PSII function and performance are limited. Despite the high O_2_ concentrations in the medium, the cells are still able to produce glycolate with high efficiency over a long period of time. As the O_2_ level is critical to the performance of the cells, it is advantageous to measure and monitor the O_2_ concentration inside the reactor. While a high O_2_ supply promotes photorespiration and glycolate production during the glycolate phase, a maximum dissolved O_2_ level of 40% should not be exceeded during the growth phase. However, in closed PBRs, high dissolved O_2_ concentrations can be reached quickly by the photosynthetic activity alone. Outgassing of O_2_ is slower than O_2_ production by photosynthesis in the growth phase. In contrast, gassing and photosynthesis during glycolate production lead to constant O_2_ levels. Furthermore, the gas inflow rates are not high enough to promote increased outgassing. Efficient reactor design and novel design concepts can facilitate the targeted outgassing [42]. Integrated airlift systems and advanced degassing units can increase oxygen mass transfer and help evacuate oxygen from the cultivation system. Here, a good reactor design with optimal control of gas flow is advantageous. Oxygen levels slightly above 30% proved to be a good value for glycolate production without influencing photosynthetic efficiency.

### Balancing with absolute measured values enables structured modelling of upscaling

Photosynthesis-based biotechnological processes can be used to fix and reduce CO_2_ from the atmosphere, which can help to fight climate change. In this study, we have successfully evaluated carbon fixation in terms of carbon use efficiency for both the initial biomass growth and the subsequent carbon fixation into glycolate as the biotechnological product. The technical conditions of the reactor design enable the determination of carbon utilisation in individual reactor units, which in themselves can be regarded as individual parts of a large-scale plant. The glycolate production rate observed in this reactor was 7.5 mM d^−1^, or 667 mg l^−1^ d^−1^, which is similar to the efficiency previously measured in small shallow reactors, where 500 mg l^−1^ d^−1^ was measured [11, 12]. These rates, together with the technical equipment of the reactor, allowed a correlation between the amount of CO_2_ inflow and the amount of carbon fixed in the product at the 1m2 reactor scale. This correlation was then extrapolated to larger scales, up to the ha range. In general, the carbon mass balances obtained showed high carbon fixation of the total CO_2_ supplied during the biomass (25%) and glycolate (97%) production phases.

Our data clearly show that carbon sequestration is much more efficient during glycolate production than during biomass formation. The low CO_2_ use efficiency of 25% during the biomass production phase is mainly due to two reasons: the changes in biomass and the CO_2_ concentration of the aeration. On one hand, the low initial biomass loading combined with the high CO_2_ influx leads to low total CO_2_ fixation rates relative to the amount of CO_2_ available. On the other hand, gassing the system with 2% CO_2_ to ensure high growth rates leads to CO_2_ surplus and thus high CO_2_ efflux rates. Thus, 75% of the CO_2_ input is lost through emission during the biomass production phase. Further enhancement of the already high growth rates by increasing the CO_2_ supply would only result in higher growth if the cells were carbon limited at the current 2% CO_2_ supply. Considering that the Q_Phar_ at high biomass concentrations is close to 100% (total absorption of incident light), it can be concluded that no CO_2_ limitation occurred during the biomass phase. As a consequence, the CO_2_ supply can be adjusted to lower concentrations, e.g., as a dynamic function with increasing biomass, to optimise the process. In the future, a fully realized circular economy regarding aeration is, therefore, mandatory to effectively capture and recycle CO_2_.

In contrast to the biomass phase, during the glycolate production phase, almost all of the supplied CO_2_ is fixed and incorporated into the product glycolate. Compared to the frequently used biomass approaches, we have used a highly sophisticated alternative method to direct the fixed carbon directly into the product. To the best of our knowledge such high rates of CO_2_ fixation in the product as achieved with the presented system have not been demonstrated before with other approaches. However, the total photosynthetic carbon fixation during the glycolate phase (8.8 mg C l^−1^ h^−1^) was only half of that in the biomass phase (14.6 mg C l^−1^ h^−1^). This is mainly due to the lower CO_2_ influx (0.2% CO_2_ compared to 2% CO_2_) required to induce glycolate production by altering the CO_2_:O_2_ ratio at Rubisco. As the fixed carbon is completely incorporated into glycolate as the product, a shortage of C in the cell metabolism is to be expected. At the same time, the overall photosynthetic capacity did not change much. Since Q_Phar_ was largely unchanged during the glycolate production phase, it is likely that the reduced CO_2_ fixation resulted in a massive electron excess in cell metabolism accompanied by a reduced consumption of NADPH and ATP. Such a high electron excess triggers two responses: the induction of photoprotective mechanisms on the one hand and photoinhibition on the other. Due to the unchanged photosynthetic capacity, it can be concluded that the protective mechanisms such as alternative electron transport pathways and increased energy dissipation are sufficient to prevent photoinhibition. This raises the question of how the cell maintains its physiological health as measured by F_V_/F_M_ (Fig. 3E). We observed a steady decrease in the previously built biomass and consequently a loss of fixed carbon of up to 5.7% (Table 1). Due to the lack of CO_2_, it is very likely that the regeneration of ribulose-1,5-bisphosphate in the Calvin cycle is strongly limited. Thus, the loss of biomass during glycolate also indicates the reductive pentose phosphate pathway is used to ensure the supply of carbon skeletons in the form of ribulose-1,5-bisphosphate at the Rubisco. Fine tuning of the CO_2_ supply could minimise this biomass loss and lead to a more stable process.

Besides environmental compatibility, the aim of intensive research for biotechnological applications has been to maximize the time-per-space yield (volumetric productivity) of biomass production or a product [11]. High volumetric productivity of biomass production in photoautotrophic reactors ranges from 15 up to 21 mg DW l^−1^ h^−1^ in *Chlamydomonas* [11, 43], depending on the setup. In contrast, with mixotrophic cultivation of *Chlamydomonas*, slightly higher biomass time-per-space yields have been reported, reaching values between 5 and 29 mg DW l^−1^ h^−1^ [44]. These biomass productivities correspond to a maximum carbon fixation of 2.5 up to 14.5 mg C l^−1^ h^−1^, assuming 50% carbon content in the biomass. In comparison, the setup used here showed a biomass volume productivity of *VP*_*C*_ = 14.6 mg C l^−1^ h^−1^ (29.2 mg DW l^−1^ h^−1^) in photoautotrophic conditions during biomass growth Table 3. Up-scaling towards an illuminated reactor area of 1 ha would result in *VP*_*C*_ = 452 kmol C ha^−1^ y^−1^ (10.9 t biomass ha^−1^ y^−1^). Assuming a continuous production cycle without glycolate production phases, where these biomass rates can be maintained for one year with artificial illumination, an annual yield of 25.5 t of biomass can be achieved. In comparison, under natural light conditions in Tuscany (Italy), the cells receive > 2 times more photons per m2 and day for eight months of operation. Within this time period, the productivity of a green algae *Tetraselmis suecica* was calculated to 36 t, assuming an average productivity of 15 g biomass m^−2^ d^−1^ [45]. Additionally, assuming very high photosynthetic efficiencies, up to 64 t ha^−1^ of microalgae biomass production have been calculated [46]. This means that real biomass production reported here is relatively close compared to the theoretically calculated rates. It can be concluded from this that an increase in the amount of light in the system presented has the potential to significantly increase the rates.

For a practical industrial application, however, the crucial parameter is the volume productivity of the actual end product. The observed high volume productivity for the product of *VP*_*Glycolate*_ is about 27.7 mg glycolate l^−1^ h^−1^, (0.66 g glycolate l^−1^ d^−1^) during the glycolate phase. This resulted in a *VP*_*Glycolate*_ of 0.36 g glycolate l^−1^ d^−1^ if calculated for an entire year, taking into account the time required for biomass phase, harvesting and cleaning. Compared to *Chlamydomonas* mass cultivation for lipid production with a volume productivity of about 0.035 up to 0.11 g lipid l^−1^ d^−1^, glycolate production showed a 3 to tenfold higher carbon fixation efficiency for the product [43, 47]. Other fast-growing green algae have been calculated to reach higher lipid volume activities of about 0.15 – 0.25 g lipid l^−1^ d^−1^ [48], however, these values are still below the glycolate production observed in this study. In addition, the volume productivity of glycolate production would also be much higher compared to other high-value products, such as astaxanthin, phycocyanin and other pharmaceuticals, since these products generally represent a much lower mass proportion of the dry weight of the biomass [49]. Taken together, glycolate production is a very efficient system, outperforming lipid production in terms of productivity per time and space.

The recorded rate of glycolate production is inherently comparable to a high rate of carbon fixation for glycolate, with *VP*_*C*_ = 8.7 mg C l^−1^ h^−1^. As CO_2_ is an important and expensive resource and will become even more so in the future, this efficient use of carbon in glycolate production reduces the economic burden of CO_2_ supply. This is even more important if the excessive CO_2_ is not recycled but emitted into the atmosphere. The high carbon utilisation rate during the glycolate production phase also contributes to the environmental sustainability of this approach, as the injected CO_2_ does not escape from the system, preventing the need for refixation. This is not the case with many other biomass-based applications that use flue gas or high levels of CO_2_ for aeration.

A more comprehensive understanding of the presented system will enable further assumptions for improvements that need to be considered in future research. In terms of carbon fixation rates, increasing CO_2_ gassing during glycolate production could improve product formation, but may lead to increased CO_2_:O_2_ ratios at the Rubisco and, therefore, reduced photorespiration rates. This can possibly be counteracted by adjusting the culture to higher light intensities so that more CO_2_ is fixed and subsequently directed into glycolate. Since the light intensity used is close to the inclination point (Ik) of photosynthesis, algae cells would face more photoinhibition and subsequent photodamage, which would be a disadvantage. As mentioned earlier, higher nitrogen supply can be used as option to increase biomass loading of the reactor. Together with increased light and CO_2_ gassing, this could stabilize the rate of photorespiration. Furthermore, higher nitrogen supply may be followed by higher protein content, especially if the amount of Rubisco is increased. This would also lead to a higher throughput of the carboxylation/oxygenation reactions, thereby increasing the productivity of the process. Future work will focus on this aspect.

Finally, the overall productivity can also be controlled by the length of the individual phases within one run. Previous work has shown that the glycolate production phase can be extended to 16 days ([11]). If the current run time for the glycolate production phase can be doubled, the annual volume productivity *VP*_*Glycolate*_ will increase from 0.36 g glycolate l^−1^ d^−1^ to 0.56 g glycolate l^−1^ d^−1^ (Additional file 1: Table S2), which impressively underpins the potential of the system.

### Energetic balance—balance is based on Q_Phar_ data

The determination of the quantum use efficiencies φC in microalgae cultivation is crucial for optimizing growth, improving cultivation systems and quantifying the photosynthetic performance. It allows for a more efficient use of resources, an increase in productivity, and the development of sustainable solutions. Using the flat panel reactor, it is possible to measure the absorbed radiation (Q_Phar_), since its specific geometric design has a defined layer thickness across its entire surface, ensuring the homogeneous distribution of both the cells and the irradiated radiation. The determination of Q_Phar_ is necessary to subsequently measure the quantum efficiency φC as photons per C fixed. A theoretical value of 8 photons per C can be calculated for φC, but it has been shown that the actual φC measured in biotechnological microalgae applications is generally much higher (Table 3) [50]. With 30 mol photons per mol C, the φC in the biomass phase is extraordinarily efficient and exactly corresponds to the values calculated in [50]. However, glycolate production shows significantly lower efficiencies, with 113 mol photons mol C^−1^. This shows that under the conditions of glycolate production, a significantly higher amount of the absorbed photons is not used for the accumulation of C in organic compounds (biomass or glycolate). Instead, the energy is dissipated, e.g., as heat or fluorescence, used for alternative electron transport pathways (e.g., Mehler reaction), for regeneration of ribulose-1,5-bisphosphate via reductive pentose phosphate pathway, or consumed by respiration. From an energetic point of view, an increase in efficiency can best be achieved by a targeted reduction of these losses. The overall good φC together with the high C fixation rates (chapter 4.3) can only occur if sufficient energy is provided to meet the demand for CO_2_ fixation. Within the presented system, the energy ultimately required to illuminate the reactor with a surface area of 1 m^2^ was measured to be 3.4 kWh. If this scale is increased to 1 ha, the annual energy demand would reach 10.3 GWh (Table 3). The average incident solar flux is between 100 and 270 W m^−2^ [51]. This corresponds to an annual available energy supply of 8.8 – 23.7 GWh, of which only 45% account for PAR [52]. These numbers show that, from an energetic perspective, a glycolate plant with the current volumetric productivity is physically limited. Only small parts of the world qualify as suitable location due to sufficient light availability. Increasing the volumetric productivity is, therefore, rarely feasible when using incident solar radiation as the light source. In addition, under natural sunlight, fluctuating light intensities significantly affect the photosynthetic activity and electron usage of different physiological pathways of microalgae [38]. Therefore, artificial illumination is a viable option to set up a glycolate production facility. Moreover, it enables a constant process of glycolate production without fluctuations in light intensity or temperature. In addition, it offers the possibility of 24-h illumination, without constraints on the orientation of reactors and installation sites.

### Economic feasibility

Taking into account the data presented, the calculation shows that even with this improved technological path, the critical level for economic production of low-cost products has not yet been reached. Additional energy would be required for various reactor operations, such as aeration and heating, and the subsequent harvesting and processing of the product. To conduct a comprehensive techno-economic analysis, it is necessary to incorporate these energy inputs. Real-world data on the operation and economic feasibility of various microalgal production systems for high-value microalgal products are available [53], but cannot be directly applied to the glycolate technology. The different production process, which focuses on continuous production while using microalgae as a catalyst to very efficiently convert CO_2_ and light into the product by minimizing biomass growth, comes with low CO_2_ aeration and unique requirements for harvesting and processing. These characteristics outperform traditional biomass production approaches and must be taken into account. However, in particular for high-volume, low-value products such as glycolate, estimated production costs can vary by a factor of 100 depending on the calculations and production process [54]. Therefore, the next step will be to evaluate the technology at the pilot-scale production level. In general, maximizing the value of a biomass producing approach needs extracting of a diverse product portfolio to facilitate economic feasibility [55, 56]. In contrast, the microalgal glycolate production system yields a very high product excretion rate in relation to the residual usable biomass and, therefore, has significant advantages in terms of feasibility.

## Conclusions

In summary, the presented system can sequester 15 t ha^−1^ y^−1^ CO_2_ in glycolate and about 20 t ha^−1^ y^−1^ CO_2_ in biomass, resulting in a total annual CO_2_ sequestration rate of 35 t ha^−1^ y^−1^ of CO_2_. It can be further concluded that photosynthesis in the biomass phase is at its maximum and cannot consume all the CO_2_ supplied, whereas during the glycolate production the added CO_2_ is completely consumed. Both biomass and glycolate production exhibit exceptional volume productivities. The total capacity of the process can be calculated to 13 t ha^−1^ y^−1^ of glycolate per year. Although the system is already set up very efficiently using artificial light with high efficiency, there are still possibilities to enhance the achieved volume productivity.

### Supplementary Information

**Additional file 1:** **Table S1.** Reactor components. **Table S2.** Theoretical volume activity during one year of production if the duration of the glycolate production phase is doubled compared to the measurements presented in the manuscript. The number of runs will be reduced to 24 runs y^−1^. **Figure S1.** Flat panel photobioreactor. **A** Schematic side view. **B** Schematic overview. **C** Photobioreactor in operation. **Figure S2.** LED emission spectrum at a light intensity of PAR = 250 µ mol photons m^−2^ s^−1^.

## Data Availability

All data generated or analyzed during this study are included in this published article and its additional files.
