# Supplementary material for: Carbon and energy balance of biotechnological glycolate production from microalgae in a pre-industrial scale flat panel photobioreactor
Source: Biotechnol Biofuels Bioprod. 2024 Mar 15;17:42. doi: 10.1186/s13068-024-02479-4 (PMC10941469; doi:10.1186/s13068-024-02479-4)
Supplement: Supplementary file 1 — Additional file 1: Table S1. Reactor components. Table S2. Theoretical volume activity during one year of production if the duration of the glycolate production phase is doubled compared to the measurements presented in the manuscript. The number of runs will be reduced to 24 runs y−1. Figure S1. Flat panel photobioreactor. A Schematic side view. B Schematic overview. C Photobioreactor in operation. Figure S2. LED emission spectrum at a light intensity of PAR = 250 µ mol photons m−2 s−1. [file 13068_2024_2479_MOESM1_ESM.docx]

Additional files

Table S1: Reactor components.

|  | No. per module | 1 m² reactor |
| --- | --- | --- |
| **Reactor** |  |  |
| Borosilicate glass panels | 3 | 9 |
|  |  |  |
| **Gas Control** |  |  |
| THERM. MFC red-y smart for Ambient Air | 1 | 3 |
| THERM. MFC red-y smart for O_2_ | 1 | 3 |
| THERM. MFC red-y smart for CO_2_ | 1 | 3 |
|  |  |  |
| **Illumination** |  |  |
| Phillips MAS LEDspot CLA D 6-50W 840 PAR20 25D | 143 | 429 |
| Dimmerpack Stairville DS-2 RF DMX | 3 | 9 |
| USB to DMX Interface USB2DMX PRO |  | 1 |
|  |  |  |
| **Cooling** |  |  |
| Plate heat exchanger Hrale B3-12-20 | 3 | 9 |
| Circulation pump Halm BUPA(N) 15-6.0 N 130 | 3 | 9 |
| Pump control system |  | 1 |
| KOH pump Ismatec Reglo ICC |  | 1 |
|  |  |  |
| **Measurement** |  |  |
| Control cabinet |  | 1 |
| pH-probe Polilyte Plus H Arc 120 | 3 | 9 |
| pO2-probe VisiFerm DO Arc 120 H0 | 3 | 9 |
| Light measurement | 1 | 3 |
| IR detector | 1 | 3 |

Table S2: Theoretical volume activity during one year of production if the duration of the glycolate production phase is doubled compared to the measurements presented in the manuscript. The number of runs will be reduced to 24 runs y^-1^.

|  | Number | volume productivity *VP_Glycolate_* | |
| --- | --- | --- | --- |
|  | of runs | [mg Glycolate l^-1^ h^-1^] | [g Glycolate l^-1^ d^-1^] |
| Measured data from the manuscript | 30 | 14.83 ± 0.61 | 0.36 ± 0,01 |
| Theoretical data of doubled production time | 24 | 23.54 ± 0.97 | 0.56 ± 0,02 |

## *Figures*


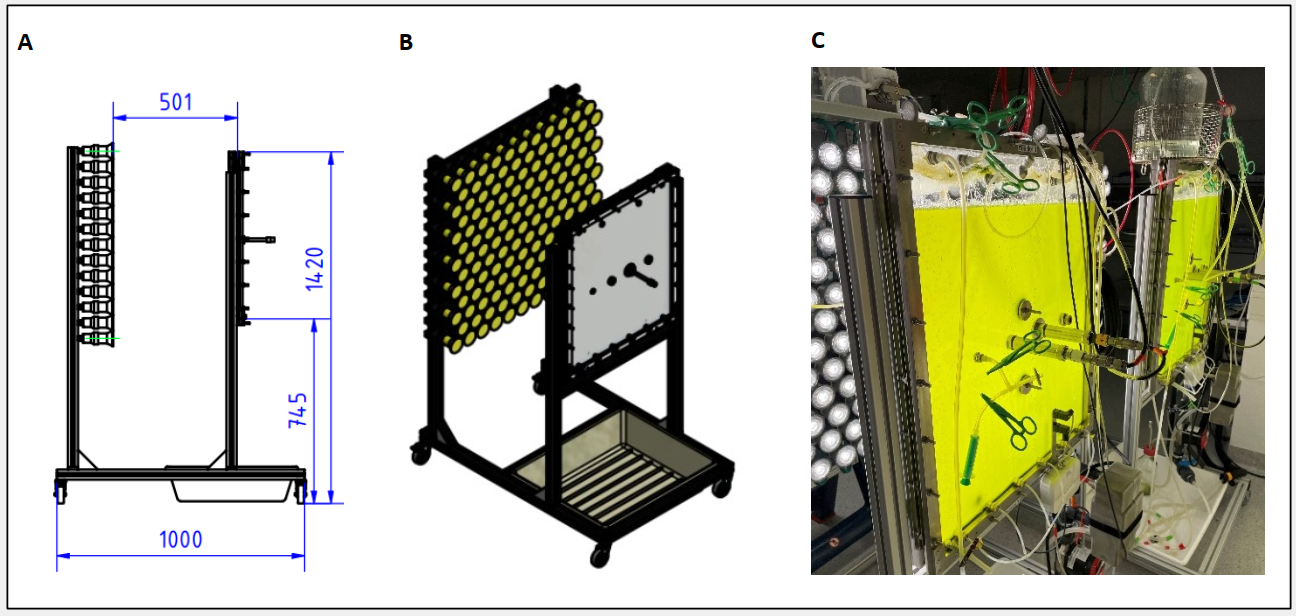


Figure S1: Flat panel photobioreactor. A: Schematic side view. B: Schematic overview. C: Photobioreactor in operation.

Figure S2: LED emission spectrum at a light intensity of PAR = 250 µmol photons m^-2^ s^-1^.
